# Targeting Heat Shock Protein 90 for the Treatment of Malignant Pheochromocytoma

**DOI:** 10.1371/journal.pone.0056083

**Published:** 2013-02-14

**Authors:** Alessio Giubellino, Carole Sourbier, Min-Jung Lee, Brad Scroggins, Petra Bullova, Michael Landau, Weiwen Ying, Len Neckers, Jane B. Trepel, Karel Pacak

**Affiliations:** 1 Program in Reproductive and Adult Endocrinology, Eunice Kennedy Shriver National Institute of Child Health & Human Development, National Institutes of Health, Bethesda, Maryland, United States of America; 2 Urologic Oncology Branch, National Cancer Institute, National Institutes of Health, Bethesda, Maryland, United States of America; 3 Medical Oncology Branch, National Cancer Institute, National Institutes of Health, Bethesda, Maryland, United States of America; 4 Synta Pharmaceuticals, Lexington, Massachusetts, United States of America; University of Missouri-Columbia, United States of America

## Abstract

Metastatic pheochromocytoma represents one of the major clinical challenges in the field of neuroendocrine oncology. Recent molecular characterization of pheochromocytoma suggests new treatment options with targeted therapies. In this study we investigated the 90 kDa heat shock protein (Hsp90) as a potential therapeutic target for advanced pheochromocytoma. Both the first generation, natural product Hsp90 inhibitor 17-allylamino-17-demethoxygeldanamycin (17-AAG, tanespimycin), and the second-generation synthetic Hsp90 inhibitor STA-9090 (ganetespib) demonstrated potent inhibition of proliferation and migration of pheochromocytoma cell lines and induced degradation of key Hsp90 clients. Furthermore, ganetespib induced dose-dependent cytotoxicity in primary pheochromocytoma cells. Using metastatic models of pheochromocytoma, we demonstrate the efficacy of 17-AAG and ganetespib in reducing metastatic burden and increasing survival. Levels of Hsp70 in plasma from the xenograft studies served as a proximal biomarker of drug treatment. Our study suggests that targeting Hsp90 may benefit patients with advanced pheochromocytoma.

## Introduction

Pheochromocytomas and paragangliomas are neural crest-derived tumors of the sympathoadrenal system. Pheochromocytomas are derived from the adrenal gland and represent a common tumor in this location in adults [1]; paragangliomas are closely-related tumors that arise from parasympathetic- and from extra-adrenal sympathetic-associated chromaffin tissues. Approximately 60–65% of pheochromocytomas are currently classified as sporadic [2]. Pheochromocytoma also presents in familial syndromes associated with germline mutations of the genes encoding *VHL, RET, NF1, SDH-A, B, C,* and *D, SDHAF2, TMEM127,* and *MAX* [2]. The majority of pheochromocytomas are non-metastatic and, when feasible, treated by surgical excision. There is no effective therapy for metastatic tumors. Among the established subtypes, tumors with SDH-B mutation have a particularly high propensity to metastasize [3]–[6].

Small numbers of patients with pheochromocytoma and paraganglioma have been evaluated for response to targeted therapy in individual case reports or as part of clinical trials of neuroendocrine tumors [7]–[9]. As yet no clinical study demonstrating positive impact of therapy on progression-free survival or overall survival has been reported in patients with metastatic disease, highlighting the importance of exploring additional molecular targets.

Heat shock protein 90 (Hsp90) is a cellular chaperone important for protein assembly, folding and stability [10]. Hsp90 is a central player in multiple pathways of cell proliferation, survival and tumor progression and consequently it has been recognized as a nodal target for cancer therapy [11]. Hsp90 is overexpressed in malignant pheochromocytoma when compared with benign disease [12], providing further support for study of Hsp90 inhibitors in this indication [13], [14]. The first clinical Hsp90 inhibitor, the benzoquinone ansamycin 17-allylamino-17-demethoxygeldanamycin (17-AAG) entered clinical trial in 1999 [15], [16]. Seventeen structurally distinct Hsp90 inhibitors have entered phase I study, including second generation, fully synthetic compounds. All of the clinical Hsp90 inhibitors bind reversibly to the N-terminal ATP binding pocket of Hsp90 and induce client protein downregulation through proteasomal degradation. These inhibitors have been extensively studied in phase I and phase II trials [11], [15], [17]. Currently no inhibitor has received regulatory approval but recent phase II studies with clinical responses that meet RECIST criteria suggest strategies for further development in several settings including HER2-positive breast cancer patients progressing on or resistant to trastuzumab, or non-small cell lung cancer patients with *ALK* mutations progressing on or resistant to crizotinib [16], [18].

STA-9090 (ganetespib) is a second generation Hsp90 inhibitor, chemically unrelated to 17-AAG and all other first generation drugs, that is more potent and potentially less toxic than 17-AAG, while maintaining similar mechanisms of action and range of indications [19]–[21].

In this article we report the use of these Hsp90 inhibitors in pheochromocytoma, evaluating cellular activity *in vitro* and efficacy in two metastatic animal models of the disease [22].

## Materials and Methods

### Ethics Statement

All animal studies were conducted in accordance with the principles and procedures outlined in the National Institutes of Health (NIH) Guide for the Care and Use of Animals and were approved by the Eunice Kennedy Shriver National Institute of Child Health and Human Development Animal Care and Use Committee,(Animal Study Proposal #12–028 and the PHS Assurance # A4149–01). Pheochromocytoma tumor tissue was obtained from two patients visiting our clinic (Institutional Review Board (IRB) protocol # 00-CH-0093) at the National Institutes of Health (NIH), in accordance with the principles and procedures outlined in the NIH IRB Guidelines, and this was approved by the Institutional Review Board of Eunice Kennedy Shriver National Institute of Child Health and Human Development (NICHD) NIH. All patients signed an IRB approved consent that allowed for the collection of tissue samples.

### Cell Line and Reagents

The mouse pheochromocytoma cell lines MPC [23] and the metastatic MPC-derived MTT cell line [24] were maintained in Dulbecco’s Modified Eagle Medium (DMEM) supplemented with 10% fetal bovine serum (FBS), 5% horse serum (Gibco, Invitrogen), and antibiotic/antimycotic. The highly metastatic MTT cells were generated by disaggregation and culture of tumor cells from a liver metastasis resected from mice inoculated with MPC cells, as described previously [24]. MTT-luc cells were generated by retroviral transduction of MTT cells with the firefly luciferase gene, as described previously [22]. The rat pheochromocytoma cell line PC12 [25] was maintained in Dulbecco’s Modified Eagle Medium (DMEM) supplemented with 10% fetal bovine serum (FBS) and antibiotic/antimycotic. The mouse and rat pheochromocytoma cell lines were grown until 80% confluence, detached using 0.05% trypsin/EDTA, and resuspended in phosphate-buffered saline (PBS) at 5×10^5^ cells/200 µl for injection. D-luciferin potassium salt (Caliper Life Sciences) was diluted in PBS at a concentration of 15 mg/ml, filter-sterilized using a 0.22 µm filter, aliquoted and stored at −20°C until use.

### Cell Migration Assay

Cell migration was measured using modified Boyden chambers (BD Biosciences). MTT cells were seeded at 150,000 cells per chamber, and cell migration was stimulated for 24 hours with serum (10%) in the absence (control) or presence of 17-AAG or ganetespib using the indicated doses. After 24 hours cells were fixed and stained using the Diff-Quick assay (Dade Behring Inc.). Mean values from four fields (1×1.4 mm) were calculated for each of triplicate wells per condition. IC_50_ values were determined using Graph Pad Prism software (Graph Pad Software). Bright field images were digitally acquired using an Olympus photomicroscope and IPLab software (Scanalytics).

### Cell Proliferation Assay

Cell proliferation was determined by 3-(4,5-dimethylthiazol-2-yl)-2,5-diphenyltetrazolium bromide assay. MTT cells (15×10^3^) were incubated in 96-well plates for 24 hours in complete medium before the addition of 17-AAG and ganetespib as indicated. A solution of 3-(4,5-dimethylthiazol-2-yl)-2,5-diphenyltetrazolium bromide (1 mg/ml; Sigma-Aldrich) was added and plates were incubated at 37°C for 3 hours before measuring absorbance at 562 nm using a Wallac Victor 3 1420 Multilabel plate reader (Perkin Elmer).

### Drug Treatment and Western Blotting

MTT cells in logarithmic growth (∼70% confluent) were treated with the indicated concentrations of 17-AAG or ganetespib for 20 hours at 37°C in 5% CO_2_. Cells were washed with cold PBS and lysed in TNESV lysis buffer (50 mM Tris-HCl pH 7.5, 2 mM EDTA, 100 mM NaCl, 1 mM sodium orthovanadate, 1% Nonidet P-40, and Complete™ (Roche) protease inhibitors cocktail). Protein lysates were denatured by boiling in 4X sodium dodecyl sulfate (SDS)-sample buffer for 5 minutes. Nuclear extracts were prepared as described by Isaacs et al. [26]. Proteins were separated by 4–20% gradient SDS-PAGE (Bio-Rad Laboratories) and transferred to a polyvinylidene fluoride (PVDF) membrane (Millipore). PhosphoS473-Akt and Akt (Cell Signaling Technology), HIF-1α (Santa Cruz Biotechnology) and tubulin (BD Pharmingen, for loading control) were measured by immunoblotting. For poly(ADP-ribose) polymerase (PARP) cleavage, MTT cells were treated with the indicated concentrations of 17-AAG or ganetespib for 20 hours at 37°C and 5% CO_2_. PARP (Cell Signaling Technology) and actin (Millipore, for loading control) were measured by immunoblotting.

### *In vivo* Studies

All animal studies were conducted in accordance to the principles and procedures outlined in the National Institutes of Health (NIH) Guide for the Care and Use of Animals and approved by the NIH Animal Care and Use Committee (ACUC). For the experimental metastasis model 5×10^5^ MTT-luc cells were injected into the tail vein of female athymic mice (Taconic Farms). For the spontaneous metastasis model 1.5×10^6^ MTT-luc cells were injected subcutaneously in the right flank of female athymic mice. Experimental groups consisted of mice injected at 10 weeks (n = 6 for 17-AAG; n = 8 for ganetespib), housed in a pathogen-free facility. The animals were imaged weekly by bioluminescence imaging (BLI) as described below. For BLI imaging, mice were anesthetized with isoflurane, injected with 150 mg/kg D-luciferin and imaged after 15 minutes as described below. For the experimental metastasis model (MTT-luc cell tail vein injection) we performed survival experiments; the endpoint criteria for euthanasia (performed by CO_2_ inhalation and cervical dislocation) included a significantly hunched posture, significantly rough fur, a body condition score (BCS) of 1 (a BCS of 3 is ideal, with 2 representing a thin condition and 1 when the animal is emaciated), or a pain score (PS) of >3 (a PS of 1 indicates no pain or distress; 2 indicates mild or transient pain or distress; and 3 and 4 indicate moderate to severe pain/distress, respectively). Conditions of the animals, including pain levels, were monitored daily.

For the spontaneous metastasis model, on day 49, after *in vivo* bioluminescence measurements were taken, the animals were euthanized (using CO_2_ inhalation and cervical dislocation), and both lungs and liver were excised and luciferase activity quantified. The organs from the mice were dissected and preserved in 10% formalin.

### Bioluminescence Imaging

All bioluminescent data were collected and analyzed with a Xenogen *in vivo* imaging system (IVIS; Calipers, Perkin Elmer). The mice were placed inside a light-tight box under continuous exposure to 1–2% isoflurane. The experiments were performed in the NIH Mouse Image Facility in accordance with ACUC regulations.

All imaging variables were equal and photographic and bioluminescent images at different time point were collected for each sample. The bioluminescence data are presented visually as a color overlay on the photographic image. Using Living Image software (Caliper, Perkin Elmer), a region of interest (ROI) was drawn around tumor sites of interest and total photons were quantified.

### Statistical Analysis

Tumor volume and mean bioluminescence was determined for each experiment together with the standard errors of the mean. Survival and other statistical analyses were performed using Graph Pad Prism software (Graph Pad Software).

### Hsp70 Secretion

Secreted Hsp70 was measured in mouse plasma by enzyme-linked immunosorbent assay (ELISA; Assay Designs Inc.), following the manufacturer’s protocol. Briefly, blood of tumor-bearing mice was collected before and 6 hours after intraperitoneal (i.p.) injection of 17-AAG as indicated. Plasma was separated by centrifugation (4°C, 14000 rpm, 10 minutes) and kept at −80°C until analysis.

### Tumor Dissociation and Tyrosine Hydroxylase Immunocytochemistry in Primary Cell Culture

Pheochromocytoma tumor tissue obtained from two patients visiting our clinic (Institutional Review Board (IRB) protocol # 00-CH-0093) was minced to 2–3 mm pieces in a 100 mm Petri dish. The pieces were transferred to 15 ml Falcon tubes filled with culture medium containing collagenase B (1 mg/ml; Roche Applied Science). The tissue pieces were incubated for 1 hour at 37°C, rocking the tube gently every 10 minutes. The pieces were allowed to settle, and the supernatant was aspirated and transferred to a new Falcon tube. The cell solution was centrifuged at 1,000 rpm/minute for 5 minutes. The pellets were resuspended in complete culture medium and the cells were seeded in 96-well plates coated with collagen IV (BD Biosciences). The following day, cells were washed with PBS and medium was added containing different concentrations of ganetespib. The final concentration of DMSO did not exceed 0.03% including control wells. The cells were treated every other day for 10 days, with drug washout and readministration every two days. On day 10, the primary cultures were washed with PBS, incubated with H_2_O_2_ and blocked for 1 hour at room temperature. The cells were then incubated with primary anti-tyrosine hydroxylase (TH) antibody (Immunostar), diluted 1∶500 in Signal Stain® Ab Diluent (Cell Signaling Technology), in humidifying chambers at 4°C overnight. The following day, cells were incubated with secondary anti-mouse-horseradish peroxidase (HRP)-conjugated antibody (Dako EnVision+ System- (HRP) Labeled Polymer; Dako) in humidifying chambers at room temperature for 1 hour. The antigen-antibody interaction was visualized using Dako Liquid diaminobenzidine (DAB)+Substrate Chromogen System. The cells were counterstained with Mayers hematoxylin solution (Sigma-Aldrich) for 5 minutes and washed in dH_2_O. Glycerol was added to prevent cells from drying and imaged immediately. TH-positive cells were counted and normalized to control (untreated) cultures.

## Results

### 17-AAG and Ganetespib Inhibit Pheochromocytoma Cell Proliferation and Migration

**Figure 1:**
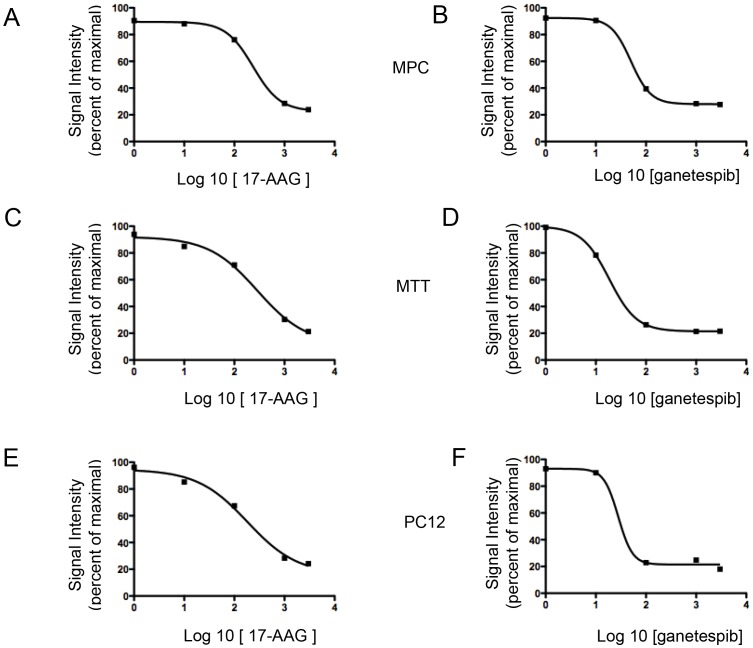
17-AAG and ganetespib inhibit cellular proliferation in pheochromocytoma cell lines. A and B) MPC cells, C and D) MTT cells, and E and F) PC12 cells were treated with different concentrations of 17-AAG or ganetespib (from 1 nM to 1 µM) and incubated for 48 hours. The graph represents the dose-response of log 10 concentrations of 17-AAG or ganetespib versus signal intensity in a thiazolyl blue formazan (MTT)-based assay.

17-AAG significantly inhibited cellular proliferation in a dose-dependent manner in all the available pheochromocytoma cell lines, namely MPC (Fig. 1A), MTT (Fig. 1C) and PC12 (Fig. 1E) cells over a range of concentrations (1 nM to 1 µM) with an IC_50_ of 235 nM, 286 nM and 181 nM, respectively. Ganetespib also caused significant inhibition of proliferation of the three pheochromocytoma cell lines and was more potent than 17-AAG, with IC_50_ values of 50 nM, 18 nM, and 28 nM in MPC (Fig. 1B), MTT (Fig. 1D), and PC12 cells (Fig. 1F).

**Figure 2:**
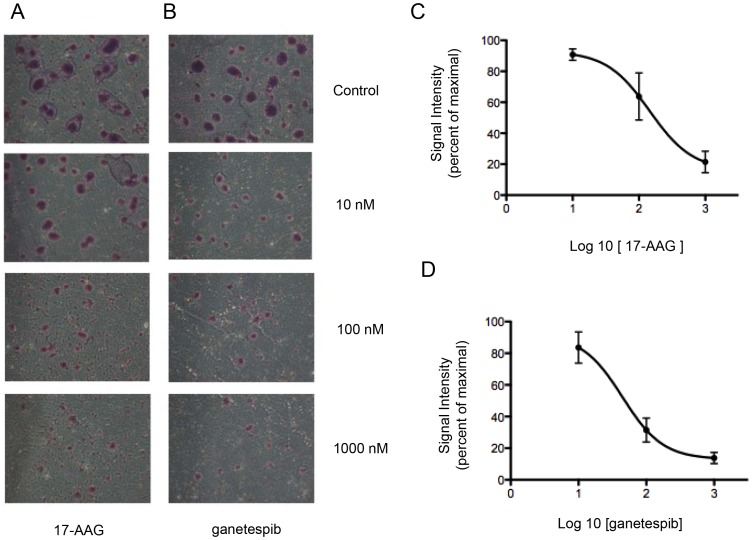
Incubation with 17-AAG or ganetespib inhibits cellular migration in pheochromocytoma MTT cells. Inhibition of migration by 17-AAG-treated (A) or ganetespib -treated (B) MTT cells in a Transwell assay in the presence of serum. Mean number of migrated cells per microscopic field (at 10× magnification) was determined from triplicate wells and is represented in this graph: 17-AAG (C) and ganetespib (D); error bars represent the standard deviation of triplicate determinations.

We tested the effect of Hsp90 inhibitor treatment on cell migration in the metastatic pheochromocytoma cell line MTT and found that 17-AAG reduced serum-stimulated migration (Fig. 2A and B), with an IC_50_ of 144 nM. Ganetespib was also able to significantly inhibit MTT migration, with an IC_50_ of 44 nM. Interestingly MTT cells tend to become confluent in clusters, reminiscent of the “zellballen” nested arrangement characteristic of pheochromocytoma histopathology. As shown in the micrograph images of Figure 2, inhibition with either Hsp90 inhibitor markedly reduced the formation of such clusters.

### 17-AAG and Ganetespib Treatment Affects Hsp90 Client Proteins in MTT Cells

**Figure 3:**
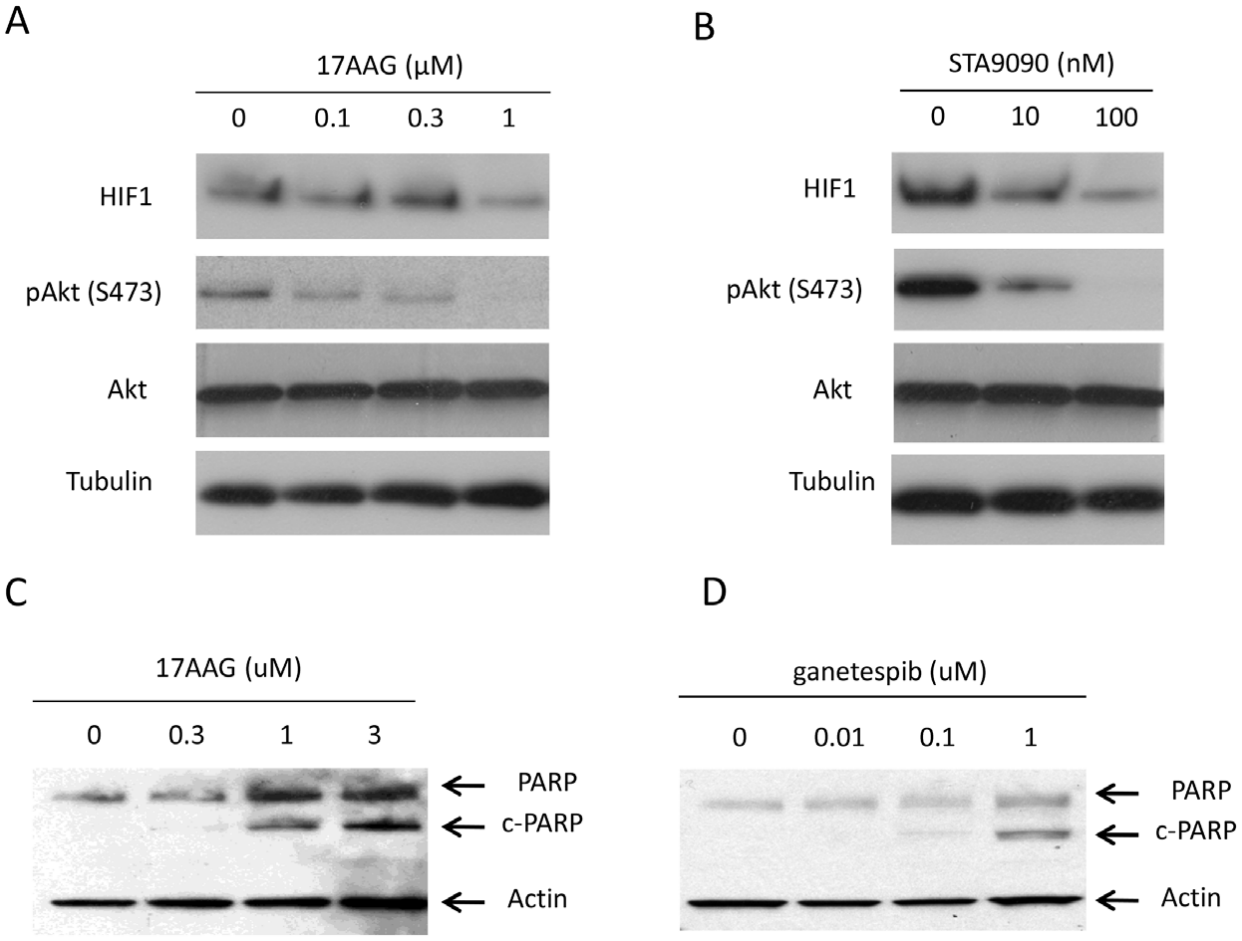
Characterization of Hsp90 client proteins affected by 17-AAG and ganetespib. MTT cells were treated with different doses of 17-AAG (A) or ganetespib (B) (0.1, 0.3, and 1 µM 17-AAG for 20 hours; 10 nM and 100 nM ganetespib for 20 hours) and total cell lysates were analyzed by western blotting. Control cells (0) were treated with DMSO vehicle alone for the same time. 17-AAG and ganetespib induce PARP cleavage in pheochromocytoma MTT cells. MTT cells were cultured with the indicated amount of 17-AAG (C) or ganetespib (D) for 20 hours and total cell lysates were subjected to western blot for PARP and reblotted for actin.

To better understand the mechanisms of cytotoxicity and growth inhibition by Hsp90 inhibitors, we treated MTT cells with different concentrations of 17-AAG (Fig. 3A) or ganetespib (Fig. 3B) for 20 hours. After treatment the levels of two established Hsp90 clients, HIF-1α and phospho-S473-Akt, were measured by western blotting. As can be seen in Figure 3, 17-AAG reduced the amount of both proteins in MTT cells. Ganetespib demonstrated a similar effect. In addition, we analyzed cleaved PARP by western blot after treatment with 17-AAG (Fig. 3C) and ganetespib (Fig. 3D) in MTT cells. Cleaved PARP increased in a dose-dependent manner, indicating that both Hsp90 inhibitors induced apoptosis in MTT cells.

### Pheochromocytoma Metastasis is Inhibited by 17-AAG and Ganetespib

**Figure 4:**
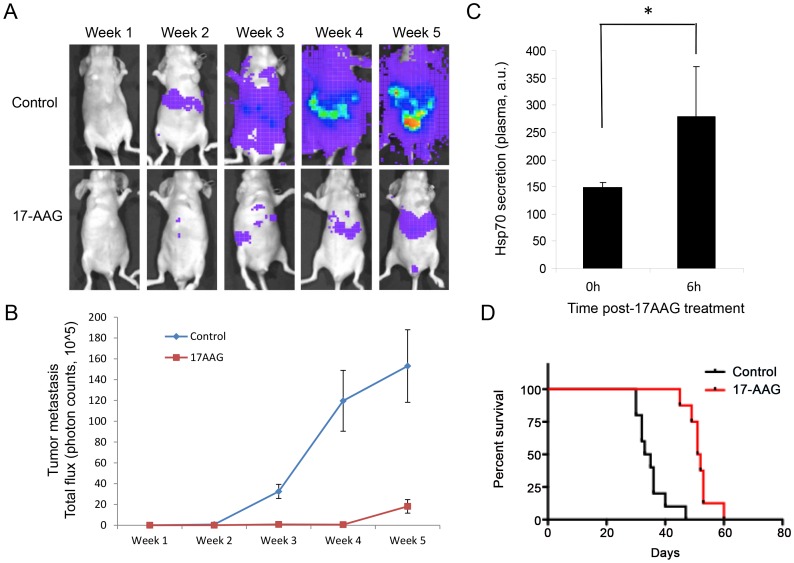
17-AAG inhibits tumor metastasis in a luciferase metastatic pheochromocytoma model. A) Efficacy of 17-AAG in an MTT-luc experimental (tail vein) metastasis model. Representative images of animals (n = 6) treated three times a week with 17-AAG over a period of 5 weeks and a control group treated with vehicle alone. B) Quantification of metastatic tumor growth as determined by luciferase measurement in an IVIS instrument. Data are plotted as mean photon counts (total flux) from regions of interest (ROI). Error bars represent standard deviations. C) 17-AAG increases Hsp70 plasma levels *in vivo* (p = 0.035). D) Kaplan-Meier survival analysis: outcome as survival time was graphed and analyzed for statistical significance using GraphPad; control (vehicle alone; black line) and 17-AAG treated animal (red line) are represented as a function of percent survival. Using a log-rank (Mantel-Cox) test the two groups were statistically different at p<0.0001.

To evaluate the effect of 17-AAG *in vivo* we employed a tail vein model of metastasis with MTT cells stably expressing the luciferase gene (MTT-luc cells). Mice were imaged immediately after injection to verify the success of the procedure (week 0, data not shown) and subsequently imaged weekly for five weeks. Tumor signals increased significantly over the six weeks of the study with exponential growth especially localized in the upper abdominal cavity (Fig. 4A, B). Eight days after tumor cell inoculation the animals were divided in two groups: one group (17-AAG, n = 6) received 60 mg/kg three times a week (i.p.); a second group (control, n = 6) received vehicle alone (DMSO) on the same schedule. 17-AAG significantly reduced the metastatic tumor burden (Fig. 4A and B) and prolonged survival (Fig. 4D, p<0.0001) when compared with the control group, with a median survival of 34 days for the control group and 51.5 days in the 17-AAG treated group.

To confirm the engagement of Hsp90 by 17-AAG *in vivo*, we measured the level of Hsp70 in plasma pre- and 6 hours post-treatment with 17-AAG. It has been reported that 17-AAG induces the synthesis of Hsp70 [16], [27] and that increases in intracellular and plasma Hsp70 can be used as a pharmacodynamic marker of Hsp90 inhibitor response [28], [29]. Thus, we measured Hsp70 levels in plasma as a biomarker for drug activity. As expected, treatment with 17-AAG increased plasma Hsp70 levels (Fig. 4C), indicative of Hsp90 inhibition *in vivo*.

**Figure 5:**
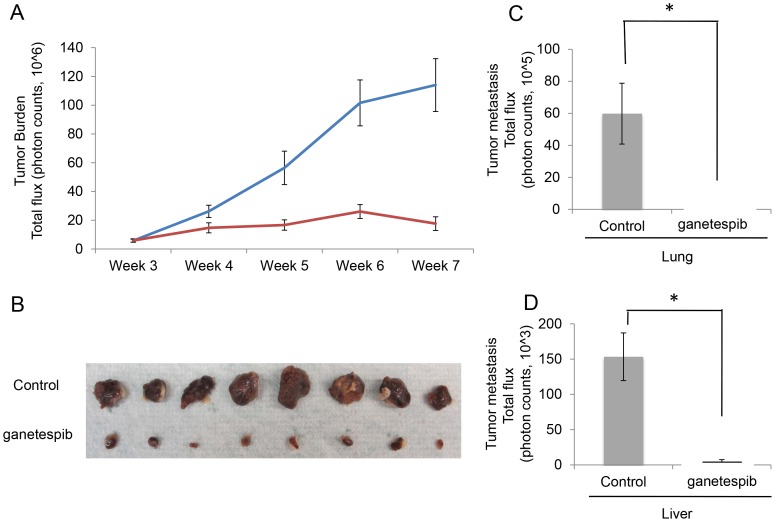
Ganetespib inhibits tumor metastasis in a spontaneous metastatic pheochromocytoma model. A) Efficacy of ganetespib in an MTT-luc subcutaneous model of pheochromocytoma. Quantification of tumor growth was determined by luciferase measurement in an IVIS instrument. Data are plotted as mean photon counts (total flux) from regions of interest (ROI). Error bars represent standard deviations. B) Primary tumors excised from the two groups were compared. C and D) *Ex vivo* bioluminescence signals from lung and liver were compared between the two groups and graphed as total flux as a method of quantification of metastatic burden at the end of the experiment (*p<0.05).

Using a spontaneous metastasis model of pheochromocytoma [22], we injected 1.5×10^6^ MTT-luc cells in the subcutaneous tissue of athymic mice. After 10 days, allowing for tumor cells to engraft, we started treatment with 20 mg/kg ganetespib, in the treated group of animals (n = 8) and the same volume of vehicle in the control group of animals (n = 8), on a daily x5, five days out of seven schedule. We then followed the growth of the subcutaneous tumor by bioluminescence detection, weekly, weeks 3 thorough 7, which demonstrated a significant reduction of tumor burden over time (Fig. 5A). As previously described [22] in the MTT pheochromocytoma model, the two main organs where spontaneous metastases develop are the lungs and livers. On day 49, after *in vivo* bioluminescence measurements were taken the animals were sacrificed and lungs and liver were excised and luciferase activity was measured by Xenogen imaging (Fig. 5C and D). Metastatic burden was dramatically reduced in lungs and livers of the treated animals compared with the vehicle control group. Primary subcutaneous tumors from each of the animals were excised and are shown in Figure 5 (Fig. 5B).

### Inhibition of Human Pheochromocytoma Cells *in vitro*

All efforts to establish cell lines from primary human pheochromocytomas and paragangliomas have been unsuccessful. Typically, tumor cells from surgical specimens can survive up to three weeks but then senesce and die [30]. To test the effect of ganetespib on human primary cell cultures, we used primary cells derived from tumor tissue collected from pheochromocytoma patients who were surgical candidates on NIH clinical protocol 00-CH-0093.

**Figure 6:**
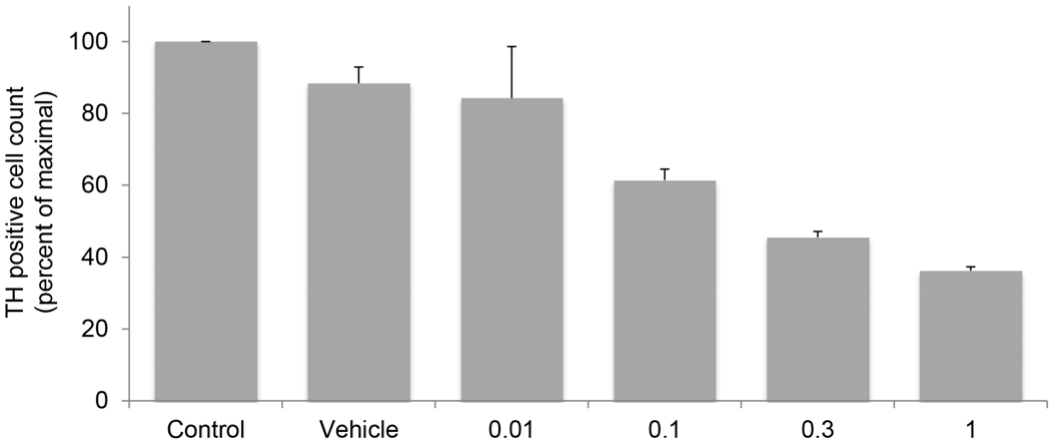
Ganetespib inhibits proliferation of primary human pheochromocytoma cells. Bar graphs represent cell counts of tyrosine hydroxylase (TH)-positive first passage primary human pheochromocytoma cells treated with ganetespib. Control indicates untreated cells; vehicle indicates DMSO treatment; the numbers under each bar indicate the ganetespib concentration (micromolar).

Immunohistochemical staining for TH, the rate-limiting enzyme in the biosynthesis of catecholamines, was used as a marker of pheochromocytoma cells to discriminate tumor from cells of the tumor microenvironment. As shown in Figure 6, treatment with ganetespib for 10 days, with drug washout and readministration every two days resulted in a dose-dependent decrease in pheochromocytoma cell number.

## Discussion

Progress in the treatment of malignant pheochromocytomas has been limited, in part because of the rarity of this malignancy, the lack of sensitivity to cytotoxic therapy, and the anecdotal nature of responses to therapy reported in small case studies or very limited numbers of patients in larger clinical trials [13], [14].

Molecular profiling studies have identified ten germline mutations that cause hereditary pheochromocytoma and paragangliomas [5], [31]. It has been suggested that these mutations can be divided into distinct molecular pathways causing errors in the HIF hypoxia-driven pathway (*VHL*, *SDHB* and *SDHD*) and errors in RNA synthesis and metabolism (*RET*, *NF1*, *MAX*, and *TMEM127*), while *KIF1BBeta* is thought to impact both pathways [32], [33]. VHL targets HIF to the proteasome under normoxic conditions and loss of VHL results in HIF overexpression and angiogenesis [32]. *SDHB* and *SDHD* mutations result in accumulation of succinate, which inhibits HIF prolyl hydroxylation, and in the absence of prolyl-hydroxylated HIF VHL cannot recognize HIF and target it for degradation, and thus HIF is overexpressed via a different but complementary mechanism from VHL mutation [2].

The genetic characterization of pheochromocytoma has indicated that targeting angiogenesis with small molecule or monoclonal antibody antiangiogenics may present a rational therapeutic strategy for metastatic pheochromocytoma patients [34]. The molecular characterization of hereditary pheochromocytoma is also supported by data indicating that VEGF is overexpressed in the majority of paragangliomas and pheochromocytomas. Moreover, pheochromocytomas and paragangliomas are often hypervascular, both radiographically and at resection [35], [36], and VEGF expression and increased microvessel density are associated with an aggressive phenotype [37], [38]. Interestingly, in a study of familial and sporadic pheochromocytomas and paragangliomas VEGF, hypoxia-inducible factor-1α and hypoxia-inducible factor-2 α (HIF-1α and HIF-2α) were prominently expressed in both tumor cells and in endothelial cells lining tumor blood vessels, and VEGF and HIF-1α and HIF-2α were more highly expressed in familial versus sporadic tumors. These data, together with transcriptional profiling analyses [39], are consistent with a pseudohypoxic angiogenic drive in the pathogenesis and progression of malignant pheochromocytoma and paraganglioma (Favier J et al Endocr Pathol 2012).

Our data showing downregulation of HIF-1α protein by Hsp90 inhibition, together with the Hsp90 inhibitor-induced cytotoxicity demonstrated here, are consistent with hypoxia as a critical adaptive response promoting survival in these tumors. In this context, Hsp90, a central player in several oncogenic signaling pathways that promote angiogenesis, tumor cell survival in low-oxygen conditions and unlimited growth, is a rational target for pheochromocytoma. Moreover, the development of inhibitors of this protein in pheochromocytoma is further supported by the knowledge that Hsp90 is overexpressed in malignant pheochromocytoma when compared with benign disease [12]–[14].

Hsp90 chaperones hundreds of intracellular proteins [40]–[42] (http://www.picard.ch/downloads/Hsp90interactors.pdf) and regulates the activity of a broad range of signal transduction pathways. Among these proteins are important elements of survival signaling networks including the PI3K/Akt pathway. In our study we have demonstrated the activity of the Hsp90 inhibitors 17-AAG and ganetespib in reducing expression of Hsp90-dependent phospho-Akt [43], [44]. It is thus of interest that levels of phospho-Akt are reported to be increased in pheochromocytoma compared to normal adrenal tissue [45].

Clinical experience has demonstrated that targeted therapies can elicit the formation of secondary mutations in target molecules, leading to formation of drug resistance [46]. The critical role of Hsp90 in the maintenance of several oncogenic drivers, represents an additional rationale for the use of Hsp90 inhibitors to eradicate cancer cells that have developed resistance to other targeted therapies.

Moreover, the capacity of Hsp90 inhibitors, such as 17-AAG and ganetespib, to interfere with several survival and resistance signaling pathways make them attractive drugs for combination therapy. Indeed, the inhibition by these drugs of multiple oncogenic signaling cascades [47] can overcome pathway redundancy and sensitize cancer cells to other chemotherapeutic compounds [48], [49].

Examination of the effect of treatment with 17-AAG in intact animals showed an increase in Hsp70 blood levels, demonstrating that the dose used in our experiment achieves biologically active plasma concentrations and affects its target. This is in agreement with other studies [28], [29]. Rajan et al. [50] have shown a statistically significant correlation of Hsp70 protein levels in peripheral blood mononuclear cells with pharmacokinetic parameters. While Hsp70 up-regulation is a clear indication of biological activity, its usefulness as a clinical biomarker for Hsp90 inhibitor therapy remains to be explored. Hsp70 is thought to promote survival of normal and tumor cells in response to cellular insults, and this prosurvival activity may contribute to therapy resistance (Wissing, Li et al. 1992). Future therapy involving Hsp90 inhibitors will be likely in combination with other therapeutics, and Hsp70 induction will provide guidance for dose selection.

Recently we developed a metastatic mouse model using highly metastatic MTT cells, which can be used for testing drugs for advanced metastatic disease [22]. We transduced MTT cells with the luciferase gene to create a stably chemiluminescent model of pheochromocytoma metastasis and employed this model for the first time here for drug development. Although other animal models of pheochromocytoma have been described (and recently reviewed by Korpershoek et al. [51]), only three have been reported to have pheochromocytoma metastasis in lungs and pelvic nerve. However our model has the advantage of reproducibly obtaining metastases in 100% of animals, and offers the advantage of monitoring tumor burden and tumor metastasis over time via non-invasive bioluminescence imaging [22]. Targeted anticancer drugs that have excellent activity *in vitro* and in subcutaneous models of *in vivo* tumor growth are rarely analyzed for activity in metastatic models, and can have markedly more limited activity or even growth-promoting activity in the metastatic milieu. The failure to test targeted therapy in orthotopic or metastatic models, as well as immunocompetent models, may contribute to late stage failure in drug development. Although the *in vivo* model used here is not in an immunocompetent host, it is a model of metastatic disease with measurable tumor growth in lung and liver, which along with bone and lymph nodes represent two of the four sites most observed in human disease. In this context, the first generation Hsp90 inhibitors have been shown to be very active against subcutaneous xenografts but to increase growth of prostatic and breast carcinoma cells in bone. In contrast, the second-generation inhibitor SNX-5422 had activity against both subcutaneous and bone tumor. Here we have used two different Hsp90 inhibitors, 17-AAG and ganetespib for *in vivo* studies, and demonstrated dramatic antitumor activity against both subcutaneous and metastatic tumor growth.

It is important to emphasize that there is not, at the moment, a human cellular model of pheochromocytoma, as every attempt to establish a continuous human cell culture from patients’ tumors has been unsuccessful. In our experiments using primary human cell cultures, we observed a dose-dependent reduction in tumor cells after treatment with ganetespib, further emphasizing the efficacy of this compound on pheochromocytoma tumor cells. In conclusion, our study supports the inclusion of pheochromocytoma and paraganglioma in the list of indications that can potentially benefit from Hsp90-directed therapy.
